# Hospital Meetings, &C.

**Published:** 1898-06-04

**Authors:** 


### HOSPITAL MEETINGS, &C.

### THE MIDDLESEX HOSPITAL.

The Hon. Walter Rothschild presided over the quarterly court of governors held on Thursday, May 26th. There were also present: Sir Arthur T. Watson, Bart., Q.C., Mr. F. Hoare, Mr. G. J. Marjoribanks, and many others. The report of the weekly board, which was adopted, stated that substantial progress had been made with the erection of the new wing for female cancer patients. The special fund for this addition had, however, been exhausted, and money was also required to repay the bankers a loan of £5,000, which was raised to meet the last quarter's tradesmen's bills. The court had, therefore, to be asked to authorise the sale, from time to time, as necessity arose, of sufficient stock to realise a total of £10,000. The board have decided to close the hospital, with the exception of the cancer ward, from July 17th to September 19th, in order that the building may be painted and other necessary repairs executed. It has been arranged to transfer the most serious cases to other hospitals, and the board have further decided to utilise two of the day rooms at the convalescent home at Clacton-on-Sea as additional dormitories for the reception of patients from the wards of the hospital. During the quarter ended March 31st 198 patients, including 14 sick nurses, were sent to the convalescent home, while 1,085 in-patients were treated at the hospital itself. In the out-patient department the number of patients was 11,322, the total attendances being 29,980, giving an average of 2 6 attendances for each patient, and an average of 329 patients seen daily. In their report the board refer to the fact that a new departure amongst the medical schools has just been initiated, nine of the larger London schools having combined to issue a joint ticket enabling qualified practitioners to attend the hospital practice at all or any of the schools, the terms being £7 7s. for three months, £10 10s. for six months, and £15s. 15s. for twelve months. The board have authorised the expenditure of £250 from the school funds for the purpose of advertising and the issue of the prospectus to the medical profession. The first floor of the new school buildings is nearly completed, and for the purpose of defraying the cost of erection the board have advanced the council of the school the sum of £2,500, at 3 per cent. interest.

### HOSPITAL FOR SICK CHILDREN.

The fifty-sixth annual court of governors of this institution was held at the hospital on Wednesday, May 25th. In the absence of the Duke of Fife, Viscount Gort presided. The annual report states that the ordinary expenditure amounted to £14,245, and the ordinary income to £10,367. Legacies to the amount of £7,057 were, however, received, making the total income £17,424, as against a total expenditure of £15,935 The committee refer with gratification to the receipt of £1,200 from the Prince of Wales's Fund, but regret that there is a falling-off of £53 in the subscriptions and £268 in the donations as compared with 1896. There is a small increase of £234 in the ordinary expenditure, which was due to a large increase in the work of the hospital. The new in patients numbered 1,946, an increase of 285 as compared with the year preceding; while the number of new out-patients was 23,673, an increase of 2,868. There were 199 patients sent to the convalescent home at Highgate, as against 240 in 1896, the decrease being due to the fact that the home was closed for ten weeks during 1897. Of the in-patients treated 673 were cured, 770 relieved, and 237 unrelieved, while 265 died. Mr. John Murray, in referring to the hospital's work, made special reference to the necessity of raising funds to cover the cost of the purchase of the property adjoining the hospital. The proposed extension of the adjoining buildings made it imperative that the property should be acquired, and a special court of governors sanctioned the purchase at a cost of £30,000. On March 1st an instalment of £10,000 was paid, but only £6,000 of this has been actually raised by contributions, so that the amount still required is £24,000. The balance of the purchase money, namely £20,000, will become due on January 1st, 1900. It is pointed out that in the buildings acquired proper accommodation for over 40 nurses will be provided. Instead of being distributed in crowded rooms in various parts of the hospital, the nurses will be nearly all lodged in one house, each nurse having a separate bedroom. There will also be dining and recreation rooms, and nearly half an acre of garden will be available for nurses and patients. Another point touched upon by Mr. Murray was the great success of the nursing home. The receipts this year have already reached the total amount received in 1896, and the 20 nurses are always out with cases. Sir Charles Crawford, Bart., moved the adoption of the report, which was carried. On the motion of Lord Aberdare, who said that one of Mr. Gladstone's characteristics was a great love of children, and that for upwards of 40 years he was a governor of this hospital, a vote of condolence with Mrs Gladstone and the dead statesman's family was unanimously passed.

### CONSUMPTION HOSPITAL, BROMPTON.

Earl Derby presided at the fifty-seventh annual meeting of the Hospital for Consumption and Diseases of the Chest, Brompton, on Thursday, May 26th. The report showed that during 1897 1,147 patients were discharged, many greatly benefited; 244 died; and there remained in the wards at the end of the year 290, the average stay of each patient being 67 days. The out-patients who received medical treatment numbered 13,098; 71,595 attendances being registered. The total ordinary income amounted to £22,693, and the expenditure to £33,627; but £22,132 had been received from legacies, enabling the Consols sold out last year to be replaced. The death of Sir Richard Quain, the senior consulting physician, who had been connected with the hospital since 1848, was mentioned with great regret, as was that of Mrs. Vertue Edwards, a former matron. It was announced that Dr. H. T. Felkin had been appointed resident medical officer, and Dr. P. S. Hichens assistant medical officer, and that a much needed home for private and staff nurses was about to be built on a site belonging to the charity in the vicinity of the hospital. Referring to the expenditure for the year, the Chairman said that some of the repairs they had undertaken were of a costly nature, and they had also carried out certain very desirable alterations. The electric light had been installed, and though they had not yet had sufficent experience of it to speak with certainty as to the comparative cost, he was convinced that even from a sanitary point of view alone the step had been a wise one. The scheme which was mooted last year for a country branch and convalescent home had, he was sorry to say, not so far received the support they had hoped for. The sum of £20,000 was wanted, but at present only £786 had been subscribed. They intended, however, to press this matter vigorously as they deemed it a most important accessory to their work. Dr. Symes Thompson emphasised the need for the country branch, as did also Dr. Theodore Williams, who pointed out that while great improvements had been mad in connection with all appliances and arrangements for alleviating chest diseases, the essential nature of fresh pure air for their successful treatment was continually becoming more evident. He was indeed, inclined to believe that it would not be long before the branch in the country would have become their main establishment. The report having been adopted the proceedings closed with the usual votes of thanks.

### NATIONAL REFUGES FOR HOMELESS AND DESTITUTE CHILDREN.

There was a crowded attendance at the fifty-fifth annual meeting of this institution, which was held at Exeter Hall on Thursday, May 26th, under the presidency of the Earl of Jersey. As a mark of respect to the memory of Mr. Gladstone, the Dead March was played on the organ at the opening of the proceedings; and in the course of his speech on the report the Chairman referred in eloquent terms to the sympathy the dead statesman had always shown for work of the character they were engaged in. A feature of the annual gatherings of this society is the presence of large numbers of the children it has rescued from a life of poverty and of degradation, and certainly the youngsters entered with keen zest into the spirit of the proceedings, whether they were giving specimens of their vocal powers or participating in the musical drill. The Chairman declared that the institution aimed at reducing the failures of humanity and increasing its successes. From its establishment to the end of 1897 it admitted 14,510 children to its refuges, and in the same period 13,710 left in order to take up the work of life. Last year 363 children were admitted, and 327 boys left for situations or to go to sea, while 46 girls were placed out in service. On December 31st last there were 787 children in the ships and homes. The subscriptions and donations for 1897 amounted to £10,635, the largest sum since 1891. A "Sixty Fund," established to celebrate Her Majesty's long reign, brought in £1,000 without any extra expense for postage or printing. The plan adopted was to ask each donor to give an amount which should represent sixty or some multiple of sixty, and the matter was taken up in the heartiest manner. In addition a subscriber sent an extra Jubilee gift of £1,020 to enable the committee to receive eighteen new boys and pay for their entire support while in the homes. The total receipts for 1897 were £19,315, and the expenditure £18,741, so that the deficit of £6,200 with which the year began was reduced to £5,626. Although those present at the annual meeting had paid for admission, a collection taken in socks knitted by the little boys in the homes realised over £63.

